# Use of Yellow Fluorescent Protein Fluorescence to Track *OPR3* Expression in *Arabidopsis Thaliana* Responses to Insect Herbivory

**DOI:** 10.3389/fpls.2019.01586

**Published:** 2019-11-29

**Authors:** Mélanie J.A. Body, Dhruveesh F. Dave, Clayton M. Coffman, Taylor Y. Paret, Abraham J. Koo, Reginald B. Cocroft, Heidi M. Appel

**Affiliations:** ^1^Division of Plant Sciences, Christopher S. Bond Life Sciences Center, University of Missouri, Columbia, MO, United States; ^2^Division of Biochemistry, University of Missouri, Columbia, MO, United States; ^3^Division of Biological Sciences, University of Missouri, Columbia, MO, United States

**Keywords:** herbivory, jasmonic acid, caterpillar feeding vibrations, OPR3, *Arabidopsis thaliana*

## Abstract

Feeding by chewing insects induces chemical defenses in plants that are regulated by the jasmonic acid (JA) pathway. Jasmonates are usually quantified by liquid chromatography–mass spectrometry (LC-MS) analysis of precursors and products in the biosynthetic pathway or inferred from the extraction and expression of genes known to respond to elevated levels of JA. Both approaches are costly and time consuming. To address these limitations, we developed a rapid reporter for the synthesis of JA based on the *OPR3*promoter:YFP-PTS1. Yellow fluorescent protein (YFP) fluorescence was increased by mechanical wounding and methyl jasmonate (MeJA) treatment and by caterpillar feeding. To develop an optimal sampling time for a quantitative bioassay, *OPR3*promoter:YFP-PTS1 plants were sampled at 1, 2, 3, and 24 h after treatment with 115 µM MeJA. The first increase in YFP fluorescence was detected at 2 h and remained elevated 3 and 24 h later; as a result, 3 h was chosen as the sampling time for a quantitative bioassay of jasmonate response to insect attack. Feeding by *Pieris rapae* caterpillars induced a 1.8-fold increase in YFP fluorescence, consistent with the known induction of JA production by this insect. We also assessed the utility of this reporter in studies of plant responses to caterpillar feeding vibrations, which are known to potentiate the JA-dependent production of chemical defenses. Pretreatment with feeding vibrations increased expression of the *OPR3*promoter:YFP-PTS1 in response to 14 µM MeJA. Feeding vibrations did not potentiate responses at higher MeJA concentrations, suggesting that potentiating effects of prior treatments can only be detected when plants are below a response threshold to the elicitor. The expression of *OPR3* does not indicate levels of specific downstream jasmonates and quantification of specific jasmonates still requires detailed analysis by LC-MS. However, *OPR3* expression does provide a rapid and inexpensive way to screen large numbers of plants for the involvement of jasmonate signaling in their response to a wide variety of treatments, and to study the induction and expression of *AtOPR3*.

## Introduction

Plant perception of insect herbivores is complex and dynamic. Leaf feeding herbivores produce multiple signals of their attack, including tissue damage, oral secretions, and feeding vibrations. Tissue damage causes increases in reactive oxygen species (ROS), oxylipins arising from damaged membranes, and calcium and electrical signaling (Savatin et al., 2014; Zebelo and Maffei, 2015; Waterman et al., 2019). Oral secretions can modulate the response to tissue damage by providing additional chemistry that alters signaling and downstream plant defense responses (Schmelz, 2015). Vibrations of the leaf caused by insect feeding can also potentiate defense responses to increase foliar and volatile defenses, independent of tissue damage and oral secretions (Appel and Cocroft, 2014; Body et al., 2019).

These local signals of insect attack can be transmitted to other parts of the leaf and other parts of the plant when they elicit internal chemical and electrical signals and external volatile signaling. For example, local tissue damage can elicit higher levels of ROS, Ca^2+^ signaling, electrical signaling, and production of jasmonates in other areas of the damaged leaf or other leaves on the plant (Miller et al., 2009; Mousavi et al., 2013; Koo, 2018; Toyota et al., 2018).

The production of higher levels of oxylipin signals like jasmonic acid (JA) and its precursor *cis*-(+)-12-oxo-phytodienoic acid (12-OPDA) is usually monitored by liquid chromatography–mass spectrometry (LC-MS) analysis of precursors and products in the biosynthetic pathway or inferred from the expression of genes known to respond to elevated levels of JA (Glauser et al., 2008; Koo et al., 2009). JA arises from the oxylipin pathway which is relatively well studied in *Arabidopsis thaliana* (Koo, 2018; Wasternack and Feussner, 2018). In brief, alpha-linolenic acid (18:3) released from galactolipid in the chloroplast membrane is converted to hydroperoxyoctadecatrienoic acid (13-HPOT) by 13-lipoxygenase. Next, allene oxide synthase and allene oxide cyclase, through sequential action, transform 13-HPOT to *cis*-(+)-12-oxo-phytodienoic acid (12-OPDA). Then, 12-OPDA is transported from the chloroplast to the peroxisome where it is converted to JA through the action of OPDA reductase (OPR) and fatty acid β-oxidation. An *OPR3*-independent pathway for JA biosynthesis has recently been discovered in transgenic plants lacking *OPR3*, but this pathway is unlikely to produce much JA when *OPR3* is present (Chini et al., 2018). Jasmonate is then released into the cytosol where it can be conjugated with the amino acid isoleucine to form JA-Ile, the ligand for the jasmonate co-receptor complex CORONATINE INSENSITIVE (COI1) (Staswick and Tiryaki, 2004; Thines et al., 2007). This co-receptor complex is key to de-repressing the jasmonate ZIM domain nuclear proteins (JAZs) that orchestrate many defense responses by integrating signals and facilitating crosstalk among the multiple hormone pathways involved in defense responses (Howe et al., 2018).

Independent of jasmonates, 12-OPDA can have activity on its own and has been hypothesized to function as a sensor and regulator of redox status (Maynard et al., 2018). More recently, 12-OPDA has been shown to be translocated from wounded shoots to roots where it is then converted into JA-Ile to regulate root JA responses (Schulze et al., 2019). As a result, levels of 12-OPDA may not be a reliable indicator of jasmonate signaling *per se*. A more reliable indicator of jasmonate activity might be possible by monitoring the expression of *OPR3*. In *A. thaliana*, there are at least three genes that code for OPR but only *AtOPR3* encodes the major enzyme active in the jasmonate pathway (Schaller et al., 2000; Chini et al., 2018). In this study, the promoter for *AtOPR3* was tagged with a yellow fluorescent protein (YFP) reporter and a PTS1 target sequence to the peroxisome to create the reporter construct *OPR3*promoter:YFP-PTS1. PTS1 was used to target the YFP to the peroxisome where *OPR3* is normally localized to allow for an increased signal intensity and sensitivity.

We developed a colorimetric YFP assay and then quantified changes in YFP concentrations in *OPR3*promoter:YFP-PTS1 transgenic plants in response to treatments with caterpillar herbivory and insect feeding vibrations. We hypothesized that *i*) YFP concentrations would increase quantitatively after feeding by caterpillars and *ii*) that insect feeding vibrations alone could potentiate the increased expression of the *OPR3*promoter:YFP-PTS1, consistent with the increase in downstream JA-related defense responses we reported previously (Appel and Cocroft, 2014; Body et al., 2019).

## Materials and Methods

### Plant Growth

*A. thaliana* (*Brassicaceae*) Col-0 wild type and *OPR3*promoter:YFP-PTS1 transgenic plants seeds were suspended in a 0.10% agar (bacteriological grade; ACROS Organics, Fisher Scientific, Hampton, NH, USA) solution and given a cold treatment at 4°C for 24 h prior to a 2-h red light treatment to trigger seed germination. The seed and agar solution were pipetted directly onto potted soil. Plants were grown in individual 2-inch circular pots (#3, 55 x 57 mm) in Pro-Mix potting soil (Premier Horticulture Inc., Quakertown, PA, USA) and enriched with 1.635 kg of Osmocote^™^ slow-release fertilizer (The Scotts Company, Marysville, OH, USA) per cubic meter of soil. The plants were grown in Percival Intellus Environmental Controller-AR66L growth chambers (Percival Scientific, Perry, IA, USA) with a photoperiod of 8:16 h (L:D), under a light intensity of 180 µmol.m^−2^.s^−1^, temperature of 22°C, and relative humidity of 62%.

#### Construction of Plasmids and Transgenic Plants

**Figure 1:**
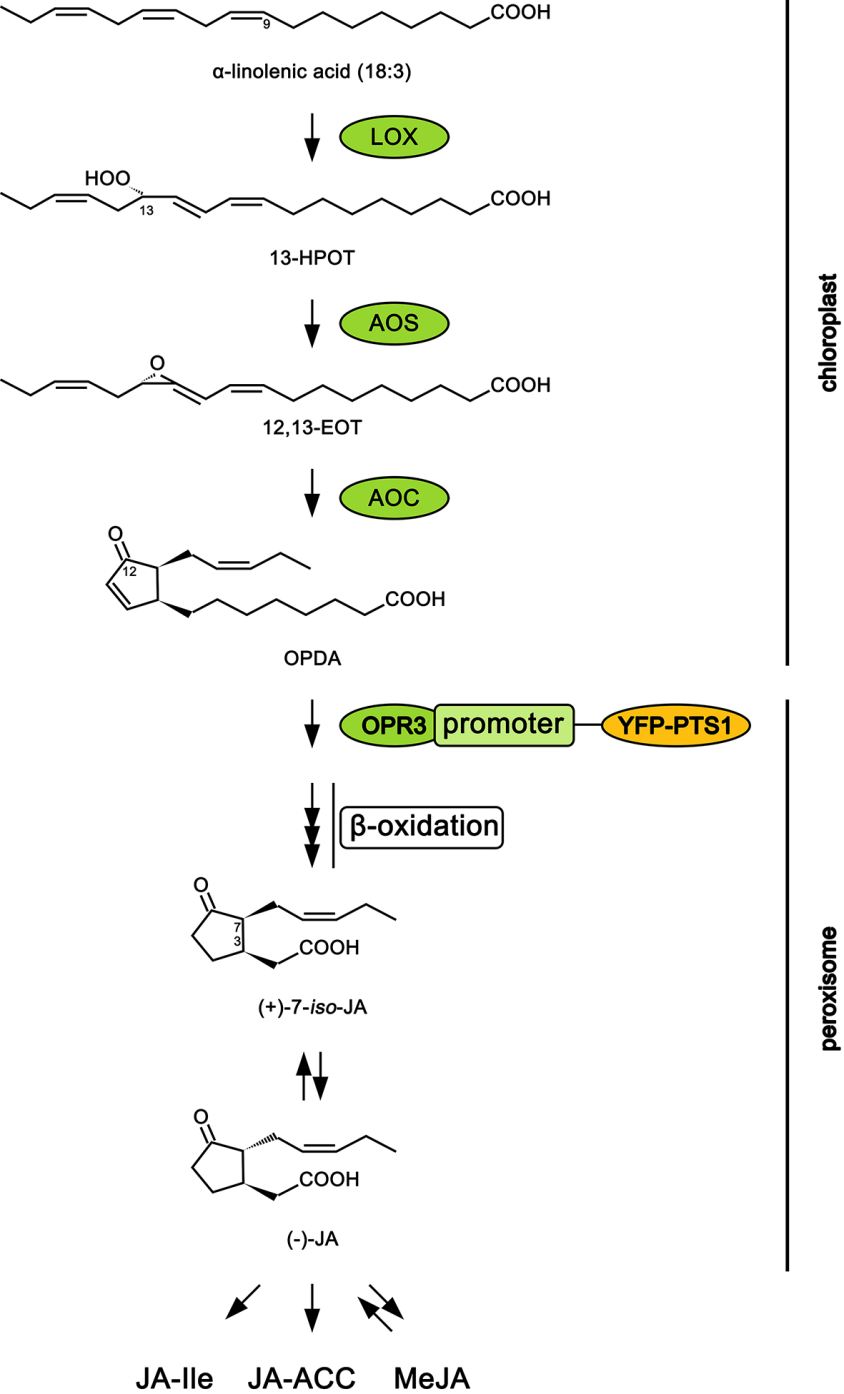
Scheme of jasmonic acid biosynthesis pathway in *Arabidopsis thaliana*. Abbreviations: 12,13-EOT, 12,13-epoxyoctadecatrienoic acid; 13-HPOT, 13S-hydroperoxy-9Z,11E,15Z-octadecatrienoic acid; AOC, allene oxide cyclase; AOS, allene oxide synthase; JA, jasmonic acid; JA-ACC, jasmonoyl-1-amino-1-cyclopropane carboxylic acid; JA-Ile, jasmonoyl-isoleucine; LOX, lipoxygenase MeJA, methyl jasmonate; 12-OPDA, 12-oxophytodienoic acid; OPR3, 12-oxophytodienoate reductase 3; YFP-PTS1, yellow fluorescent protein attached to peroxisome targeting signal type 1. Adapted from Yan et al., 2013.

The open reading frame of the gene encoding enhanced YFP (eYFP) was fused to the PTS1 peroxisomal targeting sequence (SKL; **Supplement S1**) and PCR-amplified using EYFP-Peroxi (Clontech, Palo Alto, CA) as a template using primers 5’-ATGGTGAGCAAGGGCGAG-3’ (underlined, BamHI site) and 5’-CTACAGCTTGGACTTGTAC-3’ (underlined, XhoI site). The resulting *EYFP-PTS1* was cloned into BamHI and XhoI site of a plant binary vector (pBITS) which places the gene behind the *Cauliflower mosaic virus* (CaMV) 35S promoter (*35S::EYFP-PTS1*). A 1.5 kb promoter region of the *OPR3* gene site was PCR-amplified from the *Arabidopsis* (Col-0) genomic DNA using (5’-agagtttccctgggacttggg-3’, underlined, ClaI site; 5’ATGAGAGAGATCGAATCTTCC-3’, underlined, BamHI site) primers and cloned into the ClaI and BamHI sites of *35S::EYFP-PTS1*, replacing the 35S promoter. The resulting construct was transformed into Col-0 using the floral dip method (Clough and Bent, 1998), and T_0_ seeds were screened on MS media containing 50 µg.ml^−1^ kanamycin. See **Figure 1** for the position of OPR3 in the biosynthetic pathway.

### Caterpillar Rearing

*P. rapae* (*Lepidoptera*, *Pieridae*) were reared at 24°C in 30 cm^3^ cages. Caterpillars were fed Col-0 wildtype *A. thaliana*. Butterflies were fed an 11% solution of honey (Great Value brand, Walmart, Bentonville, AR, USA) supplemented with 0.132% (wt/vol%) ascorbic acid (bacteriological grade; ACROS Organics, Fisher Scientific, Hampton, NH, USA) and allowed to oviposit on Col-0 wildtype *A. thaliana*. The caterpillars are progeny of biological stock acquired from Carolina Biological Supply (Burlington, NC, USA) and were raised for several generations under the above conditions before starting the experiment.

### Microscopy Experiment

#### Treatments

To initially visualize the effect of different treatments on YFP fluorescence, 5/6-week old *OPR3*promoter:YFP-PTS1 *A. thaliana* plants were treated at 10:00 am with one of the following treatments: wounding + MeJA 14 µM (N = 3), wounding + MeJA 115 µM (N = 3), caterpillar herbivory (N = 3), or no treatment used as a control (N = 3). For the caterpillar herbivory treatment, one post-ecdysal second/third instar *P. rapae* caterpillar was allowed to feed until approximately 30% of the target leaf area was consumed. For the wounding + MeJA treatment, a target leaf on each plant was mechanically damaged prior to the methyl jasmonate (MeJA) treatment by running a pinwheel down both sides of the midrib creating two lines of damage on each target leaf. MeJA and other jasmonate derivatives are referred to as “wounding hormones” and are commonly used to elicit chemical defense responses (Yang et al., 2015; Zhang et al., 2015; Fedderwitz et al., 2016; Lundborg et al., 2016; Tianzi et al., 2018). Plants were sprayed with either 115 µM MeJA (Sigma-Aldrich, St. Louis, MO, USA) or deionized (DI) water as a control, using an atomized mist (Flo-Master™ atomizer) for 5 s from a 45° angle, then rotated 180° and sprayed for another 5 s to ensure full coverage of the leaves.

Untreated Col-0 wild type *A. thaliana* plants (N = 1) of a similar age as the *OPR3*promoter:YFP-PTS1 plants were also used as a negative control (no YFP signal is expected in WT plants). After treatment, plants were placed in growth chambers for a 3-h incubation under the same conditions as described for plant growth.

#### Sample Preparation and Microscopy

Three hours after treatment, a 1 cm^2^ square of leaf #7 was dissected from each plant and mounted between a slide and a coverslip with a drop of DI water. Samples were imaged in the University of Toledo Advanced Microscopy and Imaging Center (AMIC; Toledo, OH, USA) using a Leica TCS SP5 inverted laser scanning confocal microscope (Leica Microsystems, Bannockburn, IL, USA) equipped with conventional solid state lasers (458, 488, 514, 561, 633 nm) and a tunable (705–980 nm) multiphoton laser (Coherent, Santa Clara, CA, USA). Images were acquired using the Leica Application Suite Advanced Fluorescence (LASAF) software at 512 x 512 in the XYZ plane in 5 µm steps with a 10X objective (NA 0.40). Specifically, YFP was excited at 488 nm and emission at 510–580 nm, chlorophyll autofluorescence was localized using excitation at 633 nm and emission at 644–714 nm. YFP signals were false-colored green and chlorophyll signals were false-colored red. 2D image projections and.avi files were made from the XYZ stacks.

### Chemistry Experiments

#### Insect Feeding Vibration Recordings and Playbacks

Vibrations produced by fourth instar *P. rapae* caterpillars feeding on leaves of potted *A. thaliana* were recorded using a laser vibrometer (Appel and Cocroft, 2014). Feeding vibrations were played back to *A. thaliana* plants for 2 h using piezoelectric stack actuators as in Appel and Cocroft (2014), with the frequency and amplitude characteristics of the playback exemplars closely matching those of the original recordings (see Michael et al., 2019 for detailed methods).

#### Experimental Designs

Experiments were conducted once on healthy 5-to-7-week old *A. thaliana* plants, and all experiments started at 10:00 am. The first experiment was conducted at the whole plant level so leaves were not numbered. For the second and third experiments, rosette leaves were numbered before each experiment using the youngest leaf larger than 6 mm as the first leaf. Leaves were numbered in ascending order corresponding to age (from young to old). On the day of the treatment, a medium leaf (usually leaf #7 or #8) was selected as a target leaf to receive the treatment. To account for potential differences in leaf age at a given position on plants of different sizes, a leaf maturity index was calculated for every target leaf by dividing its number by the total number of leaves on the plant (Coffman, 2016). The leaf maturity index was then used as a covariate in statistical analyses.

The first experiment was a time course designed to identify an optimal harvest time for the *OPR3*promoter:YFP-PTS1 genotype. Fifty *OPR3*promoter:YFP-PTS1 plants were either sprayed with 115 µM MeJA (Sigma-Aldrich, St. Louis, MO, USA) or DI water as a control. Plants were sprayed with an atomized mist (Flo-Master™ atomizer) for 5 s from a 45° angle, then rotated 180° and sprayed for another 5 s to ensure full coverage of the leaves. For each treatment, three leaves were harvested at 0, 60, 120, 180, and 1,440 min (24 h) and these were combined in a weighted average to get the value for the whole plant. A total of 49 plants per treatment was used in the experiment: 10 plants at t = 0 h, 12 plants at t = 1 h, 8 plants at t = 2 h, 10 plants at t = 3 h, 9 plants at t = 24 h. For each time period, half of the plants were treated with MeJA (four out of nine for the 24-h cohort).

The second experiment was designed to test the hypothesis that caterpillar feeding induces an increase in *OPR3* expression. Post-ecdysal second/third instar *P. rapae* caterpillars (N = 40) were removed from their host-plants and starved for 2 h before the experiment. The target leaf (leaf #7 or #8; counting down from the first fully expanded leaf that can fit in a punch hole, > 6 mm) was tagged using a paperclip cut in half and placed over the petiole with the cut ends inserted into the soil on each experimental 5 to 6-week old *A. thaliana OPR3*promoter:YFP-PTS1 transgenic plant. Half of the plants (N = 40) received one caterpillar confined *via* clip cage (foam sponge on a butterfly clip with a diameter of 3 cm and nylon mesh screening on top and bottom; Ferrieri et al., 2015) on the target leaf (#7 or #8). The other half of the plants (N = 40) had a clip cage attached to the target leaf (#7 or #8) without a caterpillar, to control for any effect of the clip cage itself. Insects were allowed to feed until approximately 30% of the target leaf area was consumed. Every time a treatment was terminated for the caterpillar treatment, the clip cage was removed from a control plant as well. After treatment, plants were placed in growth chambers under the same conditions as previously described for a 3-h incubation. Three hours after the treatments, the target leaf was harvested and weighed (fresh weight) before being flash frozen in liquid nitrogen in 1.2 ml Thermo Scientific™ Abgene™ blank and alphanumeric storage tubes (Thermo Fisher Scientific, Waltham, MA, USA).

The third experiment was designed to determine whether the expression of *OPR3* is sensitive to priming by caterpillar feeding vibrations, and if so, at what MeJA concentration the effect is evident. For each plant (N = 64), a vibration playback actuator or sham was attached with dental wax to the target leaf, as in Appel and Cocroft (2014). After 2 h of vibration playback, plants were treated with 0, 14, 29, or 58 µM MeJA as described above. After treatment, plants were placed in growth chambers under the same conditions described above, for a 3-h incubation. Three hours after the treatments, the target leaf was harvested along with a younger leaf and an older leaf from the same orthostichy, i.e., adjacent to the left and right of the target leaf. Leaf age was included in the statistical model to allow us to assess systemic responses (Appel and Cocroft, 2014). Leaves were harvested and weighed (fresh weight) before being flash frozen in liquid nitrogen as described above.

#### Sample Storage and Preparation

Samples from all experiments were stored in a −80°C until dried in a lyophilizer (Genesis 25 SQ Super ES; VirTis—SP Scientific, Gardiner, NY, USA) for 6 h under the following conditions: −40°C for 2 h, 0°C for 2 h, 40°C for 2 h and held at 22°C until removed. The pressure in the lyophilizer was kept below 400 mTorr. Lyophilized samples were placed in resealable plastic bags with desiccant (t.h.e.(R) Desiccant (Indicating) 8% 8 Mesh; Millipore Sigma, Billerica, MA, USA). Then, they were weighed (dry weight) on a microbalance (Mettler AE 100 Analytical Balance; Mettler Toledo, Columbus, OH, USA), ground to a fine powder for 5 min using a MBB-96 Mini-BeadBeater (Biospec Products Inc, Bartlesville, OK, USA) and then centrifuged for 2 min at 5,000 x *g* before chemical analysis.

#### Chlorophyll Extraction

Chlorophyll was removed from leaf tissue with a chloroform/DI water wash by adding 513 µl chloroform and 537 µl DI water to the leaf powder. Cluster tubes were capped and inverted 100 times by hand to allow for chlorophyll extraction, then centrifuged for 2 min at 5,000 x *g*. At this stage, the bottom layer contains the chlorophyll (green), while the top aqueous layer contains the YFP (clear).

#### Yellow Fluorescent Protein Quantification

Three aliquots of 130 µl from the top layer of each sample were pipetted into black 96-well microplates (Thermo Fisher Scientific, Waltham, MA, USA) as three technical replicates. To collect an accurate measurement of the fluorescence, each well was given 300 flashes at 514 nm (excitation) and read at 529 nm (emission) using a PerkinElmer EnSpire 2300 Multilabel Reader (PerkinElmer, Waltham, MA, USA).

#### Calibration Curve

Fluorescein is not very soluble in water, therefore a stock 50 mM (16.62 mg/ml) fluorescein solution was prepared using a 100 mM sodium carbonate solution. The stock solution was used to prepare the fluorescein standard working solution at 20 µM and the serial dilution to establish a standard curve allowing the conversion of fluorescence to concentration. One hundred and thirty microliters of fluorescein standard were pipetted at the following concentrations: 0, 14, 28, 42, 56, 70, 84, and 98 µg/ml onto each 96-well microplates.

### Statistical Analysis

Statistical analyses for all three chemistry experiments were conducted in SAS v. 9.4 (PROC GLIMMIX). A general linear mixed model (GLMM) was performed using a lognormal or gamma distribution. For experiments 1 and 2 (testing the effect of harvest time and caterpillar feeding), “round” (the set of plants tested at the same time) was included as a random effect. For experiment 3 (testing the effect of feeding vibrations), “plant” was included as a random effect to account for including measurements of three leaves per individual, and the relative maturity of the harvested leaves (leaf maturity index) was included as a covariate. This experiment consisted of a 2 × 2 design (MeJA +/−, vibration +/−) run at four MeJA concentrations. To simplify the interpretation of the results and avoid the need for comparisons among eight treatment combinations, we analyzed the data separately for each MeJA concentration. For *post hoc* analyses, p-values were adjusted using the false discovery rate procedure of Benjamini and Hochberg (1995) to account for multiple comparisons. All YFP fluorescence emission are expressed as emission at 529 nm per mg of dry weight (DW) of leaf tissue (least squares mean ± standard error).

## Results

### Visualization of Yellow Fluorescent Protein Fluorescence

**Figure 2:**
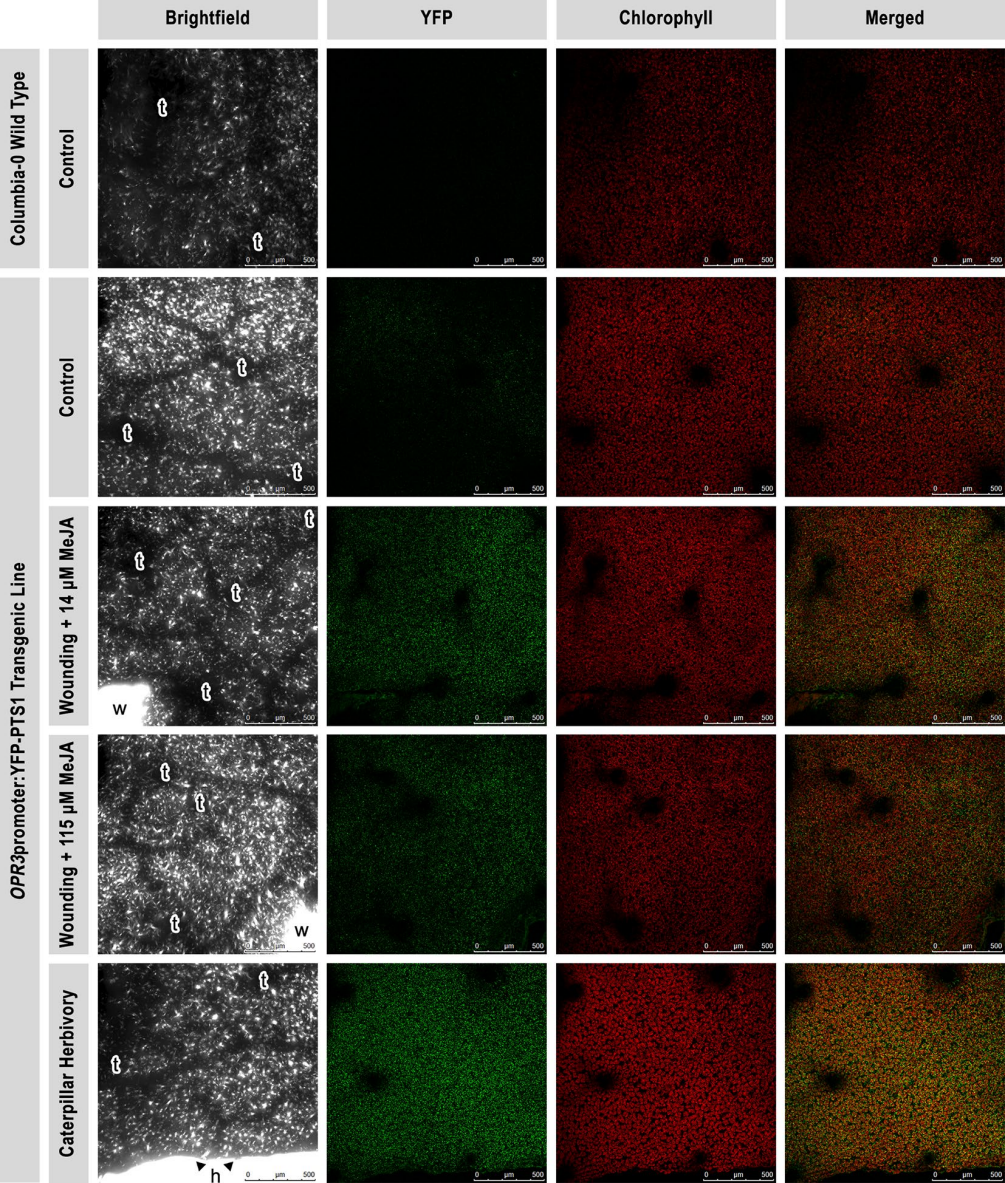
Visualization of the transient expression of *OPR3*promoter-YFP-PTS1 fusion in *Arabidopsis thaliana* leaves. Expression and localization of *OPR3*, the gene encoding the enzyme that converts 12-oxo-phytodienoic acid to jasmonic acid, using confocal laser scanning microscopy. Brightfield (column 1), yellow fluorescent protein (YFP) fluorescence (green; column 2), chlorophyll autofluorescence (red; column 3), and a merged image of the two channels are shown (column 4). A wild type *A. thaliana* plant (N = 1; row 1) was used as a negative control to show the absence of autofluorescence in the YFP wavelength range (no signal). *A. thaliana OPR3*promoter:YFP-PTS1 transgenic plants were used as a control (N = 3; row 2) for the reporter construct to show the basal *OPR3* level (low signal) of intact and healthy plants. *A. thaliana OPR3*promoter:YFP-PTS1 transgenic plants were submitted to one of the three following treatments to show an increase in *OPR3* levels after a 3-h incubation: wounding + 14 µM MeJA (exogenous plant-derived chemical elicitor; N = 3; row 3), wounding + 115 µM MeJA (N = 3; row 4), and *Pieris rapae* caterpillar (larval instar L2-L3) herbivory (N = 3; row 5). Leaf #7 (target leaf) was imaged on all of the plants. A single image for each treatment is shown but is representative of all three images per treatment. All micrographs were taken at 10x (zoom 1.0), scale bar = 500 µm. *t*, trichome; *w*, wound; *h*, herbivory.

A confocal laser scanning microscope was used to visualize the transient expression of YFP fused to the promoter of the gene for the peroxisomal enzyme (**Supplement S1**) REDUCTASE 3 (OPR3), *OPR3*promoter:YFP-PTS1, in *A. thaliana* (**Figure 1**). OPR3 is involved in the conversion of 12-OPDA (JA precursor) into JA, a reaction essential for production of bioactive jasmonates that promote plant defense against wounding and insect herbivory (Koo et al., 2009; Koo, 2018). Wild type plants showed no YFP fluorescence consistent with their lack of the YFP-*OPR3* fusion (**Figure 2**, row 1). Control *OPR3*promoter:YFP-PTS1 transgenic plants showed a low signal, reflecting the basal expression of *OPR3* in healthy plants (**Figure 2**, row 2). In contrast, plants that were mechanically wounded and sprayed with MeJA (exogenous plant-derived chemical elicitor), or fed upon by *P. rapae* caterpillars displayed an elevated YFP fluorescence, reflecting a higher level of *OPR3* expression, and therefore the activation of the JA pathway (**Figure 2**, rows 3, 4, 5). Wounding + MeJA (14 µM) triggered a higher plant response than wounding + MeJA (115 µM) (**Figure 2**, rows 3, 4), suggesting that a higher concentration does not necessarily lead to a higher plant response. Compared with MeJA spray, regardless of the concentration, caterpillar herbivory triggered the greatest YFP fluorescence increase, compared with untreated control plants (**Figure 2**, row 5). Caterpillar herbivory also seemed to induce an increase in chlorophyll fluorescence, an indication of increased rates of photosynthesis.

### Time Course of Yellow Fluorescent Protein Fluorescence in Response to Methyl Jasmonate Spray

**Figure 3:**
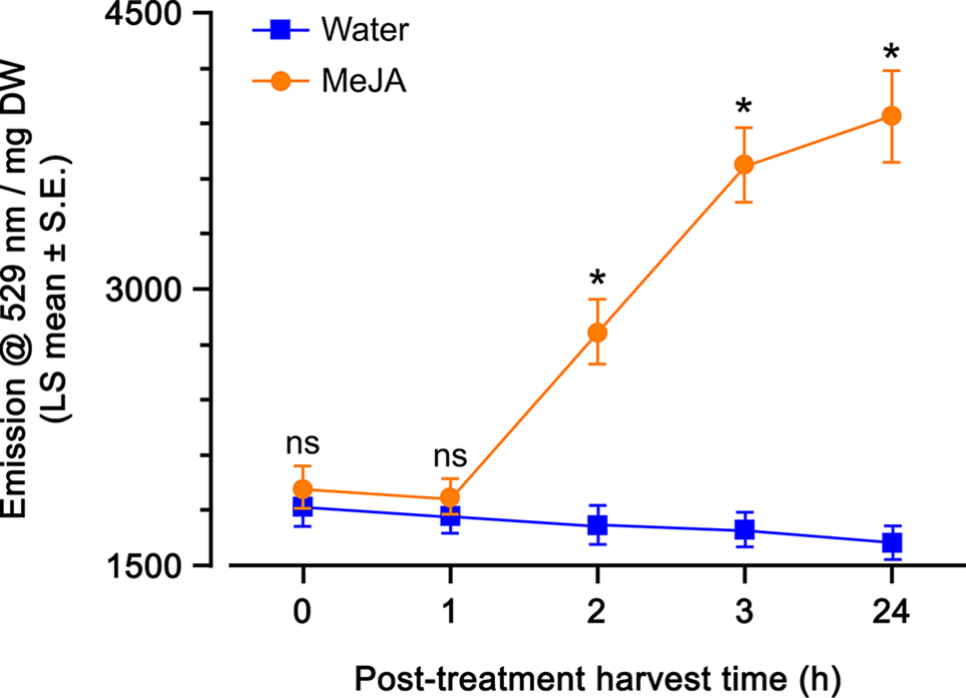
Yellow fluorescent protein (YFP) fluorescence emission (emission at 529 nm per mg dry weight; least squares mean ± standard error) of *Arabidopsis thaliana OPR3*promoter:YFP-PTS1 plants over time after methyl jasmonate (MeJA) spray compared with water spray. Statistical differences (p-value ≤ 0.05), based on a general linear mixed model, between water and MeJA spray at each time are shown by asterisks; ns, non-significant. N = 10 plants at t = 0 h, N = 12 plants at t = 1 h, N = 8 plants at t = 2 h, N = 10 plants at t = 3 h, N = 9 plants at t = 24 h per treatment. See **Table 1** for statistical details.

A time course of MeJA spray (115 µM) was used to determine what incubation time was the most appropriate to monitor YFP fluorescence in *A. thaliana OPR3*promoter:YFP-PTS1 transgenic plants. There was a highly significant direct effect of MeJA and incubation time on YFP fluorescence, and a highly significant interaction of these two factors (**Table 1**). Plants sprayed with just water had a stable YFP fluorescence, regardless of the incubation time, indicating that the spray itself did not induce a plant response in the JA-pathway (**Figure 3**). Within 1 h after spray treatment, there was no difference in YFP fluorescence of plants sprayed with water and with MeJA (**Figure 3**). However, after 2-, 3-, and 24-h incubation times, YFP fluorescence was 1.5-fold higher in plants treated with MeJA spray compared to plants treated with water spray (**Figure 3**). The YFP fluorescence at 2, 3, and 24-h incubation times did not differ from one another (**Figure 3**); therefore, for convenience, we decided to use a 3-h incubation time for the subsequent experiments.

**Table 1:** Effect of incubation time after methyl jasmonate spray on yellow fluorescent protein fluorescence emission of all aboveground tissue, based on a general linear mixed model.

Treatment	d.*f*.	F value	Pr > F
MeJA	1, 39	158.51	0.000 ***
Incubation time	4, 39	15.91	0.000 ***
MeJA × incubation time	4, 39	24.97	0.000 ***

Statistical differences are shown by asterisks (***) when p-value ≤ 0.001. N = 49 plants. d.f., degree of freedom; F value, F statistic value; Pr > F, probability of the F statistic.

### Induction of Yellow Fluorescent Protein Fluorescence by *Pieris rapae* Caterpillar Herbivory

**Figure 4:**
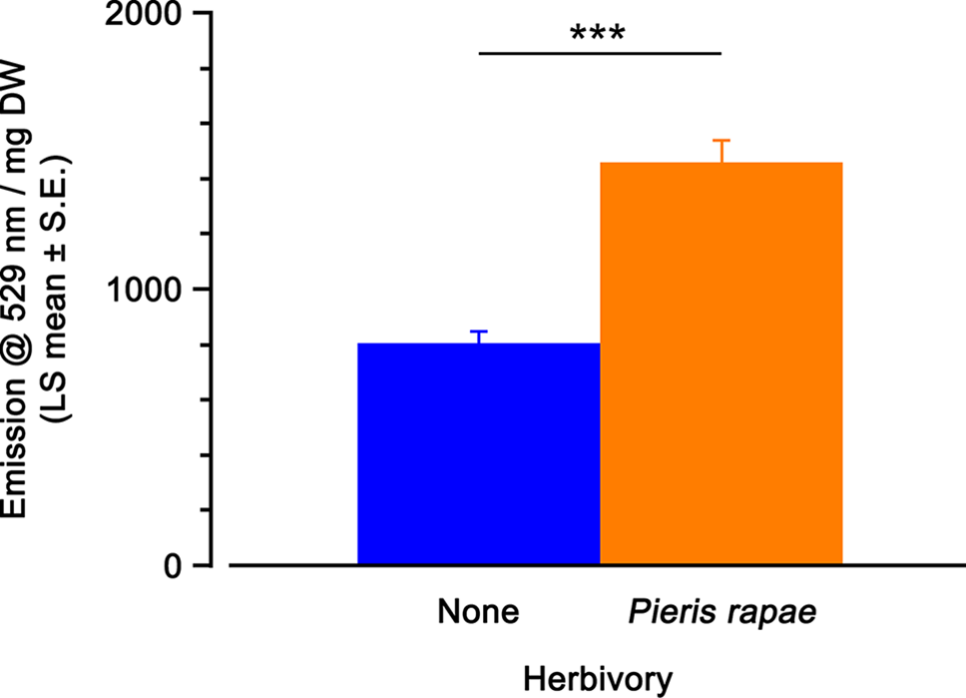
Yellow fluorescent protein (YFP) fluorescence emission (emission at 529 nm per mg dry weight; least squares mean ± standard error) of *Arabidopsis thaliana OPR3*promoter:YFP-PTS1 plants after *Pieris rapae* caterpillar herbivory, compared with control plants. Statistical differences (p-value ≤ 0.001), based on a general linear mixed model, between different treatments are shown by asterisks. See **Table 2** for statistical details. N = 40 per treatment.

Herbivory had a significant effect on YFP fluorescence on leaves of *A. thaliana OPR3*promoter:YFP-PTS1 plants (**Table 2**). Feeding by individual second/third instar *P. rapae* caterpillars until approximately 30% of the leaf area was removed led to a 1.8-fold increase in YFP fluorescence, compared with the fluorescence level of undamaged *OPR3*promoter:YFP-PTS1 plants (**Figure 4**). Since in this experiment individual leaves and not whole plants were used, a leaf maturity index (Coffman, 2016) was calculated to account for potential differences in leaf age at a given position on plants of different sizes. There was no significant effect of leaf maturity index on YFP fluorescence (**Table 2**).

**Table 2:** Effect of *Pieris rapae* herbivory on yellow fluorescent protein fluorescence emission, based on a general linear mixed model.

Treatment	d.*f*.	F value	Pr > F
Herbivory	1, 76	61.86	0.000 ***
Leaf maturity index	1, 76	1.48	0.228

Statistical differences are shown by asterisks (***) when p-value ≤ 0.001. N = 80 plants. d.f., degree of freedom; F value, F statistic value; Pr > F, probability of the F statistic.

### Induction of Yellow Fluorescent Protein Fluorescence by Insect Feeding Vibrations

The effect of insect feeding vibrations on the response of *A. thaliana OPR*3promoter:YFP-PTS1 plants to a range of MeJA concentrations was measured. YFP fluorescence of the vibrated leaf, a younger leaf, and an older leaf from the same orthostichy was measured at each MeJA level. At three of the MeJA concentrations (0, 29, 58 µM) there was no significant effect of vibration, leaf age, vibration × leaf age, or leaf maturity index on YFP fluorescence (**Table 3**). However, at 14 µM MeJA, there was a significant effect of vibration and leaf age on YFP fluorescence with no significant effect of vibration × leaf age and leaf maturity index (**Table 3**).

**Table 3:** Effect of insect feeding vibration compared with silent sham, at different methyl jasmonate concentrations, on yellow fluorescent protein fluorescence emission, based on a general linear mixed model.

MeJA concentration	Treatment	d.*f*.	F value	Pr > F
	Vibration	1, 24	0.25	0.623
0 µM	Leaf age	2, 24	1.76	0.194
	Vibration × leaf age	2, 24	0.30	0.745
	Leaf maturity index	1, 24	0.28	0.603
	Vibration	1, 25	6.10	0.021*
14 µM	Leaf age	2, 25	4.23	0.026*
	Vibration × leaf age	2, 25	1.24	0.307
	Leaf maturity index	1, 25	2.50	0.127
	Vibration	1, 24	0.10	0.749
29 µM	Leaf age	2, 24	1.10	0.350
	Vibration × leaf age	2, 24	0.31	0.734
	Leaf maturity index	1, 24	0.08	0.775
	Vibration	1, 20	1.72	0.205
58 µM	Leaf age	2, 20	0.77	0.476
	Vibration × leaf age	2, 20	1.96	0.167
	Leaf maturity index	1, 20	0.02	0.886

Statistical differences are shown by asterisks (*) when p-value ≤ 0.05. N = 64 plants. d.f., degree of freedom; F value, F statistic value; Pr > F, probability of the F statistic. Note that “leaf age” is an identifier for the three harvested leaves (target, older, younger).

**Figure 5:**
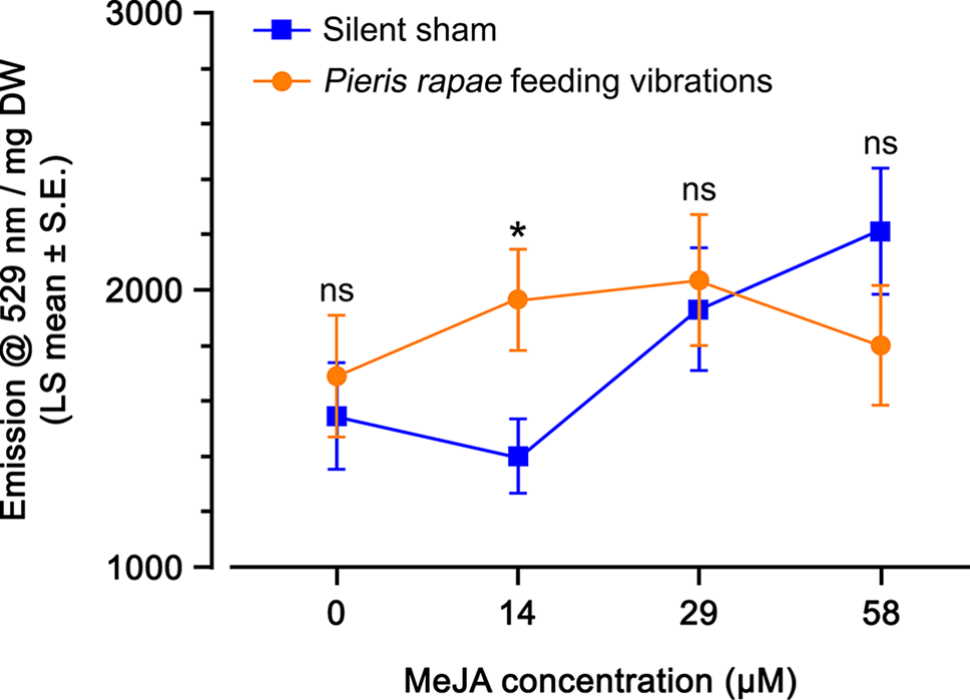
Yellow fluorescent protein (YFP) fluorescence emission (emission at 529 nm per mg dry weight; least squares mean ± standard error) of *Arabidopsis thaliana OPR3*promoter:YFP-PTS1 plants after priming with insect feeding vibrations, in response to different methyl jasmonate (MeJA) concentrations, compared with a silent sham. Statistical differences (p-value ≤ 0.05), based on a general linear mixed model, between the silent sham and insect feeding vibration for each MeJA concentration are shown by asterisks; ns, non-significant. See **Table 3** for statistical details. N = 8 per treatment.

Plants sprayed with higher MeJA concentrations (29 and 58 µM) had levels of YFP fluorescence 1.5-fold higher than plants sprayed with lower MeJA concentrations (0 and 14 µM MeJA) (**Figure 5**). There was no difference between the YFP fluorescence of plants that received insect feeding vibrations compared with plants that were subjected to the silent sham except at 14 µM MeJA where the YFP fluorescence in the silent sham had not yet started to increase (**Figure 5**). Similar to what we observed in the microscopy experiment, the detection of a priming effect of insect feeding vibrations is possible only when the plant’s response to MeJA is not at its maximum. Taken together, these results suggest that there was no direct effect of insect feeding vibration on the activation of JA-pathway (water spray), but we observed a priming effect of the vibrations when plants were treated with 14 µM MeJA.

## Discussion

We developed a quick and inexpensive method to quantify YFP expression in leaves of *A. thaliana* plants with an *OPR3*promoter:YFP-PTS1 reporter and showed that it was induced by MeJA and caterpillar feeding within 1 h. This is consistent with other work with *A. thaliana,* in which the expression of *OPR3* is induced by mechanical wounding, MeJA, insect feeding, and/or insect oral secretions (Costa et al., 2000; Koo et al., 2009; Leon-Reyes et al., 2010; Koo et al., 2011). Thus, we can monitor the involvement of jasmonate signaling in a defense response using *OPR3* expression as a proxy.

The utility of a given reporter depends on the question being asked. For localization, GUS, GFP, and YFP are all useful; however, these reporters are not customarily used for quantitative assays. Our development of a quantitative assay for a YFP reporter could also be used for GFP reporters.

The expression of *OPR3* does not indicate levels of specific downstream jasmonates since it does not account for any post-translational modification or *OPR3* protein stability and turnover. Quantification of specific jasmonates still requires detailed analysis by LC-MS. However, *OPR3* expression does provide a rapid and inexpensive way to screen large numbers of plants for the involvement of jasmonate signaling in their response to a wide variety of treatments, and to study the induction and expression of *AtOPR3*.

The quantitative method we developed does not lend itself readily to measurement at shorter time periods after treatment even though expression of *OPR3* could be rapid. YFP expression at earlier time points may be obtained through non-quantitative localization of *OPR3* gene expression using fluorescent microscopy or stereomicroscopy.

Using *OPR3* expression, we showed that caterpillar feeding vibrations in the absence of the actual caterpillar potentiated the impact of MeJA on OPR3 promoter activity, causing an increase in YFP fluorescence compared to MeJA treatment alone. This result is consistent with our previous work in which 2 h of caterpillar feeding vibrations primed the phenolic and glucosinolate defense responses of *A. thaliana,* both known to involve JA signaling (Appel and Cocroft, 2014). In a previous experiment measuring phytohormones, we found that levels of 12-OPDA and JA were increased by 24 h of insect feeding vibrations when accompanied by MeJA with mechanical wounding; in the absence of MeJA treatment, levels of 12-OPDA, JA, and JA-Ile were lowered by feeding vibrations (Body et al., 2019). Although substrate-borne caterpillar feeding vibrations differ markedly from the airborne tones used in most studies of plant responses to “sound,” single airborne tones can also lower the levels of JA (Ghosh et al., 2016). In addition, *A. thaliana* responses to touch also involve JA signaling (Chehab et al., 2012), although we do not know yet how these very different stimuli compare. In experimental contexts, they differ greatly in the duration and intensity of mechanical stimuli and tissue treated.

Our observation that the *OPR3* response was saturable at high concentrations of MeJA suggests that there is an upper limit of plant response to MeJA. This is not surprising, but it has important implications for the design of experiments to detect the effects of a prior treatment. For example, we saw a potentiation effect of insect feeding vibrations only at a lower MeJA concentration. If experiments to detect effects of prior treatments are not designed to elicit responses below the upper limit of the response variable, the effects of a prior treatment on that response will be difficult to demonstrate.

How insect feeding vibrations interact with other early signaling components that also upregulate the JA pathway is an important next step in understanding how these multiple signals interact to produce an adaptive plant response to herbivory. JA is present in only trace amounts in undamaged leaves but within minutes accumulates locally and systemically in damaged leaves. This is preceded by release of plant and herbivore derived oligosaccharides and peptides, generation of ROS, changes in cellular pH, extracellular nucleotides, and/or changes in plasma transmembrane potential (reviewed in Maffei et al., 2007 and Koo, 2018). Whether insect feeding vibrations directly activate JA biosynthesis through their hypothesized perception by mechanoreceptors or elicit secondary messengers remains to be determined. The development of reporters to localize the action of multiple signal components over time will be key to understanding their interaction in producing a coordinated adaptive response.

## Data Availability Statement

All datasets generated for this study are included in the article/**Supplementary Material**.

## Author Contributions

AK made the *OPR3*promoter:YFP-PTS1 reporter construct and the transgenic lines. DD and CC designed the protocol for the colorimetric assay to quantify YFP. MB, DD, and TP ran the experiments. RC and CC performed the statistical analyses. MB, DD, and HA wrote the manuscript. All coauthors reviewed the manuscript.

## Funding

This project was supported by NSF IOS-1359593 to HA and RC, and NSF IOS-1557439 to AK.

## Conflict of Interest

The authors declare that the research was conducted in the absence of any commercial or financial relationships that could be construed as a potential conflict of interest.
