# Supplementary material for: Use of Yellow Fluorescent Protein Fluorescence to Track OPR3 Expression in Arabidopsis Thaliana Responses to Insect Herbivory
Source: Front Plant Sci. 2019 Nov 29;10:1586. doi: 10.3389/fpls.2019.01586 (PMC6897264; doi:10.3389/fpls.2019.01586)
Supplement: Supplementary file 1 [file DataSheet_1.pdf]

Supplement S1. MeJA-inducibility and subcellular localization of *OPR3*promoter:YFP-PTS1 in *Arabidopsis* leaves. Confocal microscopy image of transgenic *Arabidopsis* leaves carrying *OPR3*promoter:YFP-PTS1 treated with mock (water with < 0.01 % (v/v) ethanol) (left) or 50 mM MeJA (middle) for 12 h. The characteristic green punctate structures that are indicative of peroxisomes are seen in MeJA-treated *OPR3*promoter:YFP-PTS1 lines similar to the control peroxisomal marker YFP-OPLC1 (right; from Koo et al. 2006). Red-colored oval structures are chloroplasts. Images represent a projection of ten optical sections (1 μm depth/section). Scale bars = 20 μm.

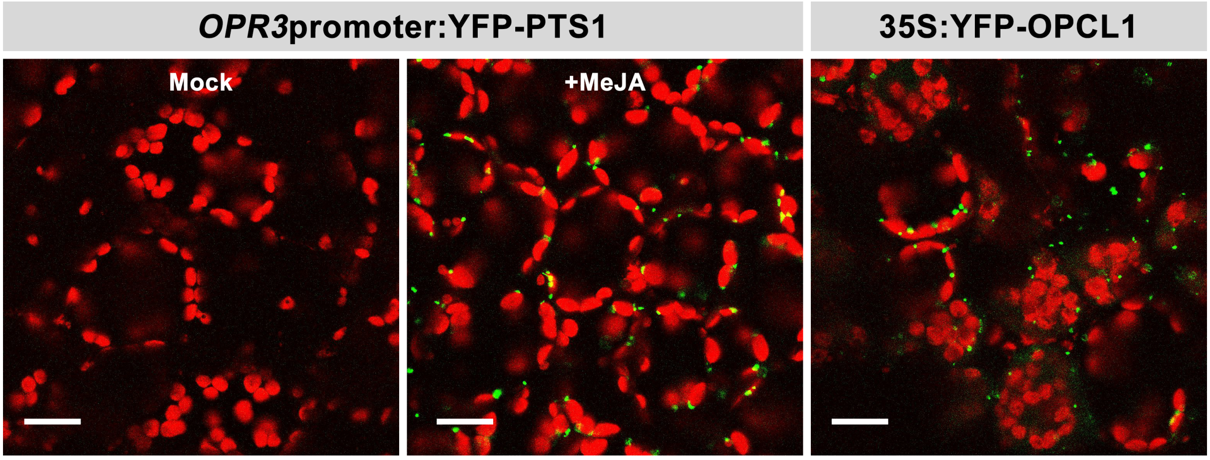

Koo, A.J.K., Chung, H.S., Kobayashi, Y., and Howe, G.A (2006). Identification of a peroxisomal acyl-activating enzyme involved in the biosynthesis of jasmonic acid in *Arabidopsis*. *J. Biol. Chem.* 281, 33511-33520. doi: 10.1074/jbc.M607854200
